# Noise-robustness test for ultrasound breast nodule neural network models as medical devices

**DOI:** 10.3389/fonc.2023.1177225

**Published:** 2023-06-22

**Authors:** Jiaxin Jiang, Xiaoya Jiang, Lei Xu, Yan Zhang, Yuwen Zheng, Dexing Kong

**Affiliations:** ^1^ School of Mathematical Sciences, Zhejiang University, Hangzhou, China; ^2^ Department of Mathematics, The Hong Kong University of Science and Technology, Clear Water Bay, Kowloon, Hong Kong SAR, China; ^3^ Zhejiang Demetics Medical Technology Co., Ltd, Hangzhou, China; ^4^ Department of Interventional Therapy, The Hwa Mei Hospital of the University of Chinese Academy of Sciences, Ningbo, China

**Keywords:** breast ultrasound, breast nodule, robustness, deep learning, image noise, network performance analysis

## Abstract

**Background:**

Deep learning technology has been widely applied to medical image analysis. But due to the limitations of its own imaging principle, ultrasound image has the disadvantages of low resolution and high Speckle Noise density, which not only hinder the diagnosis of patients’ conditions but also affect the extraction of ultrasound image features by computer technology.

**Objective:**

In this study, we investigate the robustness of deep convolutional neural network (CNN) for classification, segmentation, and target detection of breast ultrasound image through random Salt & Pepper Noise and Gaussian Noise.

**Methods:**

We trained and validated 9 CNN architectures in 8617 breast ultrasound images, but tested the models with noisy test set. Then, we trained and validated 9 CNN architectures with different levels of noise in these breast ultrasound images, and tested the models with noisy test set. Diseases of each breast ultrasound image in our dataset were annotated and voted by three sonographers based on their malignancy suspiciousness. we use evaluation indexes to evaluate the robustness of the neural network algorithm respectively.

**Results:**

There is a moderate to high impact (The accuracy of the model decreased by about 5%-40%) on model accuracy when Salt and Pepper Noise, Speckle Noise, or Gaussian Noise is introduced to the images respectively. Consequently, DenseNet, UNet++ and Yolov5 were selected as the most robust model based on the selected index. When any two of these three kinds of noise are introduced into the image at the same time, the accuracy of the model will be greatly affected.

**Conclusions:**

Our experimental results reveal new insights: The variation trend of accuracy with the noise level in Each network used for classification tasks and object detection tasks has some unique characteristics. This finding provides us with a method to reveal the black-box architecture of computer-aided diagnosis (CAD) systems. On the other hand, the purpose of this study is to explore the impact of adding noise directly to the image on the performance of neural networks, which is different from the existing articles on robustness in the field of medical image processing. Consequently, it provides a new way to evaluate the robustness of CAD systems in the future.

## 1 Introduction

According to statistics, Ultrasound scanners now produce billions of diagnostic images each year (1). However, the large amount of medical image data brings a heavy task to the radiologists. In order to solve this clinical problem, many computer-aided diagnosis (CAD) schemes have been developed, aiming to help clinicians more efficiently read medical images and make the diagnostic decision in a more accurate and objective manner (2).

Breast cancer is now the most common cancer in the world. It is the first leading cause of cancer mortality in women worldwide with an estimated 2.26 million cases recorded in 2020. Nearly 685,000 of these women died of breast cancer. Almost two-thirds of those deaths were recorded in less-developed regions (3). Breast ultrasonography is one of the most widely used methods to evaluate a subject’s breast, because it has the advantages of non-invasive, fast and repeatable (4). Researchers developed computer-aided diagnosis (CAD) algorithms for Breast cancer screening because of the substantial presence of breast ultrasound data in hospitals. CAD system based on deep neural network has the potential to assist clinicians with ultrasonic diagnostic tasks. At present, the basic application of CAD system in ultrasound image is segmentation, detection and classification. The purpose of ultrasound image segmentation is to separate the ROI region from the unimportant background. The purpose of the classification task is to determine whether breast tumors are benign or malignant. The detection task of ultrasound image is to obtain the specific location of the lesion area based on the classification results. In recent years, popular deep learning methods have widely been recognized as a reliable approach, because of the good data interpretability as well as the high discriminable power. For breast ultrasound image classification, Yaozhong Luo et al. proposed a human–machine knowledge aggregation network based on channel attention to fuse features from images and human descriptions to classify breast tumors (5). Their method can effectively improve classification performance. Runyi Li et al. proposed the Efficientnet model based on the cbam attention mechanism, and added a multi-scale fusion method (6). The results contribute to the further study of breast cancer staging.For segmentation tasks, unlike traditional segmentation methods which mainly rely on low-level image features, Qinghua Huang et al. propose a novel segmentation method with semantic classification of superpixels for BUS images (7). This is Inspired by the use of local receptive field in the CNN model. Meng Lou et al. improving the U-shaped CNN through adaptively reducing semantic gaps and enhancing contextual relationships between encoder and decoder features, thereby achieving fully automated semantic segmentation in ultrasound imaging (8). On the other hand, Yaozhong Luo et al. focused on the relationship between two vision tasks tumor region segmentation and classification and proposed a novel segmentation-to-classification scheme by adding the segmentation-based attention (SBA) information to the deep convolution network (DCNN) for breast tumors classification (9). It is worth noting that deep convolutional neural network (CNN) has also made significant progress in object detection (10–12). Due to the development on deep learning techniques, CAD system have managed to obtain accuracies comparable to or even exceeded human-level experts’ performances (13–15). This diagnostic accuracy boost is mainly the result of advances in two directions, namely, building more powerful multi-layer neural network architecture and collecting more clinical data. Some Breast cancer screening tools based deep neural networks have already received market approval.

Despite these CAD systems have been remarkably successful in a variety of medical imaging tasks to support disease detection and diagnosis, their robustness has not been thoroughly studied. Recent studies in recognition of pigmented skin lesions found that minor-scale perturbations can have an effect on the robustness of AI diagnostic algorithm (16). Ryoungwoo Jang et al. had validated the deep learning–based computer-aided diagnosis model for binary classification of posteroanterior chest x-ray through random incorrect labeling in 3 datasets. They found computer-aided diagnosis with inaccurate labels is not credible (17). Chenyang Shen et al. showed that little noise perturbations could have an effect on the robustness of a deep neural network that classify CT images of lung nodules into malignant or benign groups (18). They found that models with different architectures and parameters have different robustness concern, and they proposed an adaptive training scheme to solve this problem. For precise and accurate diagnosis, medical images need to be clear and free of noise and artefacts. In fact, as these medical imaging technologies thrive to achieve high resolution images, they end up packing increasing number of pixels per unit area (19). This makes the images more susceptible to noise. Compared with other medical images, ultrasound images have worse resolution and significant noise with a low signal-to-noise ratio. At the same time, in order to obtain better image contrast in the process of ultrasound imaging, specific TGC(Time Gain Control) will be set. These Settings also introduce new noise. Therefore, it has important clinical value to evaluate the stability of AI-assisted diagnostic system against noise.

Despite the increasing use of DCNN in breast ultrasound diagnosis, there are still some problems to be addressed. There have been few studies on the effects of using low-quality ultrasound images of the breast for diagnosis. The lack of research investment in this area has created uncertainty when noisy images are presented for CAD systems applications. The significance of this research is the bridging of this uncertainty when comparing the performance of different techniques. In this work, we evaluate the robustness of a CAD system by adding different levels of noise to the test set and training set respectively and observe the changes in CAD system accuracy. Specifically, we compare the robustness of 9 (including three for image classification, three for target detection, and three for image segmentation) network structures commonly used in CAD systems by injecting different kinds of noise with different concentrations into the input images. And observe the effects of noise on the 9 neural network outputs. Interestingly, we find that the networks of the same architecture share similar robustness properties. And the variation trend of accuracy with the noise level in Each network used for classification tasks and object detection tasks has some unique characteristics. This finding provides us with a method to reveal the black-box architecture of CAD systems. On the other hand, the purpose of this study is to explore the impact of adding noise directly to the image on the performance of neural networks, which is different from the existing articles on robustness in the field of medical image processing. At present, most scholars study the robustness of CAD systems to adversarial noise, which makes them have to know the detailed network structure of CAD systems. In this paper, we can evaluate the robustness of CAD systems without knowing the network structure.

## 2 Materials and methods

### 2.1 Dataset

Breast ultrasound images can produce great results in the classification, detection, and segmentation of breast cancer when combined with machine learning (20). In this study, collected from Ningbo No.2 Hospital of Zhejiang Province in 2021 and 2022, our training dataset contains a total of 8617 images with different sizes. And each breast ultrasound image label and lesion area are annotated and voted by three sonographers based on their malignancy suspiciousness to reduce the error among them. We used these 8617 breast ultrasound images to train the nine neural networks mentioned above, including 4443 benign cases and 4174 malignant as shown in **Table 1**.

**Table 1:** Composition of experimental data.

Distribution of images	Training Set	Test Set
Number of benign images	4443	487
Number of malignant images	4174	210
Number of total images	8617	487

The Dataset used in the test experiment is an open-source Dataset: Dataset of Breast Ultrasound Images (20). The dataset is based on 780 breast ultrasound images collected in 2018 among women between the ages of 25 and 75. The size of the PNG images in the dataset is 500*500 pixels. The ground truth images are presented with original images (20). The breast ultrasound dataset we used as a test set was categorized into two classes, which are 487 benign images and 210 malignant images.

### 2.2 Methods

**Figure 1:**
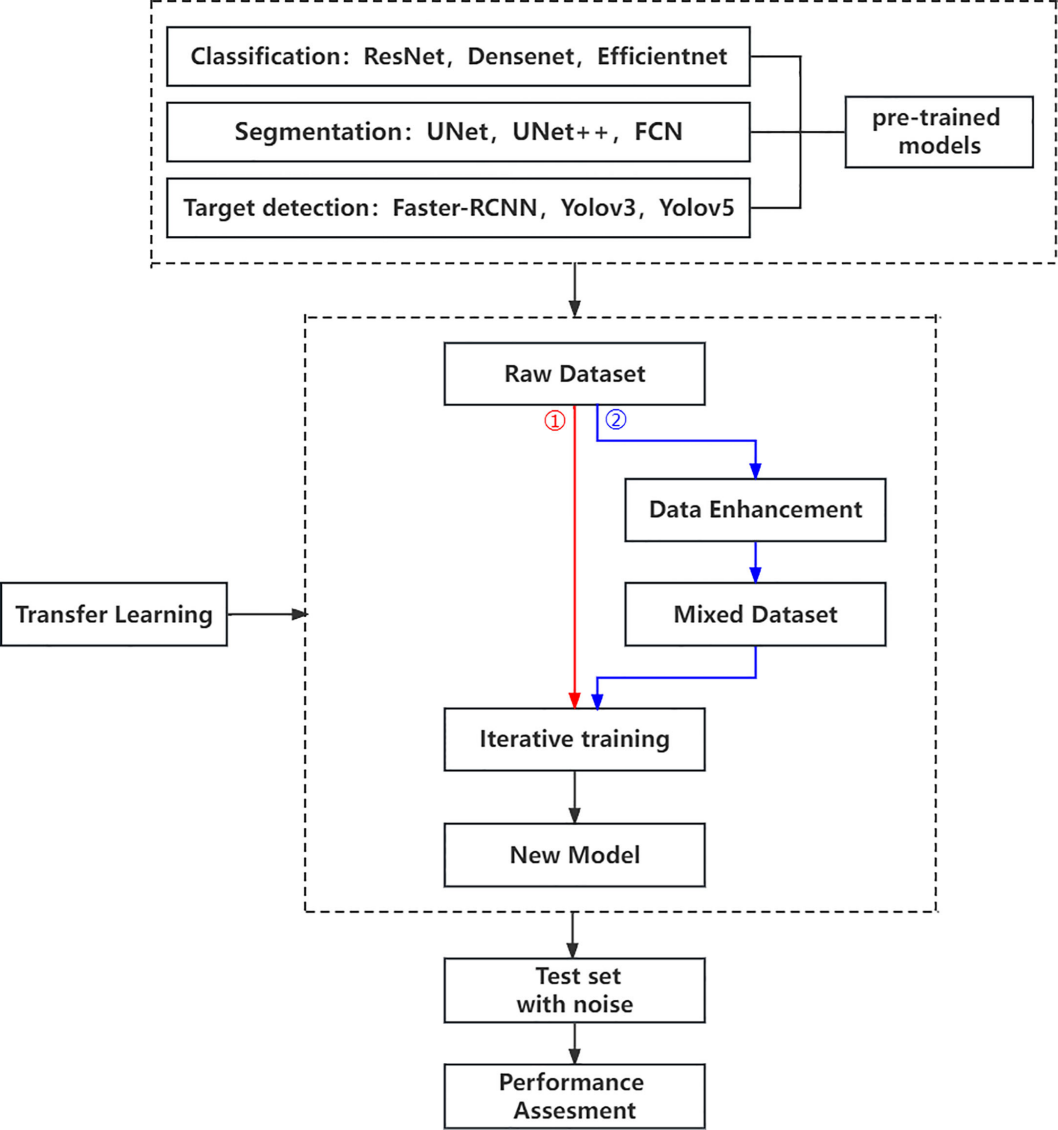
Research workflow.

**Figure 2:**
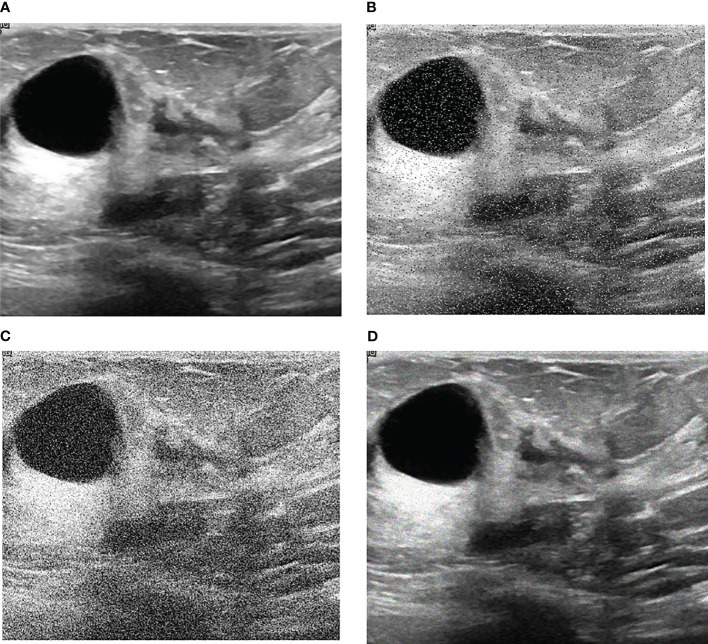
Noise impact on breast ultrasound images. **(A)** Original Image. **(B)** Salt & Pepper Noise. **(C)** Gaussian Noise. **(D)** Speckle Noise.

Our research is based on the workflow depicted in the **Figure 1** below. Firstly, we used 8617 breast ultrasound images to train nine neural networks introduced in Section 2.3 and evaluated their performance on this benchmark. On the other hand, the part of data enhancement was added in the training. Manoj Gupta et al. explored the various kinds of noises present within the ultrasound medical images and also the filters that are used for the noise removal purpose. They found that the noises were introduced in the ultrasound images are Salt and Pepper Noise, Speckle Noise, Gaussian Noise and Poisson noise (21). For this problem, we choose three kinds of noise, namely Salt and Pepper Noise, Gaussian Noise and Speckle Noise, to directly add to the picture. Noise was added to 60% of the 8617 images, with random intensity Salt and Pepper Noise added to 20% of the images, random intensity Gaussian Noise added to 20% of the images, and random intensity Speckle Noise added to the rest. Afterward, three types of noise, including Gaussian Noise, Salt and Pepper Noise and Speckle Noise, are applied to the test set separately. The ratio of pepper noise to salt noise is fixed at 1:1. The addition level of each noise is 0, 0.02, 0.04,0.06, and so on. For Salt and Pepper Noise, the noise level is the ratio of Salt and Pepper Noise points added in this experiment to replace the noise points in the original figure. And for Gaussian Noise, the level of added noise is controlled by adjusting the size of the standard deviation of the Gaussian distribution. The larger the standard deviation is, the more noise will be added, and the more severely the image will be damaged. Due to the Speckle Noise does not follow normal distribution and quite close to Rayleigh and Gamma distributions. For Speckle Noise, the level of added noise is controlled by adjusting the size of the standard deviation of the Gamma distribution. The ultrasound image of the breast with three kinds of noise added and the original image are shown in **Figure 2**.

Then, we use evaluation indexes in 2.4 to evaluate the robustness of the neural network algorithm respectively. On the other hand, to establish the models’ susceptibility to image changes, each algorithm was evaluated on the training set that added the same kind of noise as mentioned above. Of course, we apply the same noises for the test sets and evaluate the robustness of the neural network algorithm respectively. Finally, some conclusions for evaluating the robustness of CAD systems are summarized.

### 2.3 Neural network architectures

In this work, we study the elementary knowledge of 3 image classification algorithms, 3 image segmentation algorithms, and 3 object detection algorithms, as summarized below. The Implementation of neural networks that we use in our experiments are all publicly available.

The ResNet (Residual Neural Network) we used is a 50-layer network of the model introduced by He Kaiming et al. (13). ResNet uses a skip connection with an identity transformation (13) which could effectively alleviate the problem of gradient explosion and gradient disappearance. Thus, good performance can be guaranteed to train a deeper network. The DenseNet (Dense Convolutional Network) we used is a 34-layer network by Huang, G et al. (22). The direct connection from any layer to all subsequent layers in the DenseNet model. This way can effectively alleviate the problem of gradient disappearance, reduce a lot of parameters (22) and improve the information flow between different layers. In addition, DenseNet can enhance the propagation of feature maps to compare with ResNet (23). The EfficientNet we used is the basic version of the network designed by Tan Mingxing et al. (24). When designing and scaling a convolutional neural network, EfficientNet employs a compound coefficient (24) to scale all dimensions of depth, breadth, and resolution. This can greatly improve the performance of convolutional neural networks.

FCN is a fully convolutional neural network that can take input of arbitrary size and produce correspondingly-sized output with efficient inference and learning (25). Since FCN uses no fully connected layers, it reduces the number of network parameters to a large extent, thus FCN can simplify and speed up network learning. In the field of medical image processing, UNet is a widely used neural network proposed by Ronneberger (26). As a widely recognized variant of FCN, UNet has the advantage of shortening paths and skipping connections to enhance detailed information (26). UNet++ is the enhanced version of UNet by Zhou Z (27). The significant improvement of UNet++ over the classical U-Net architecture is ascribed to the advantages offered by the redesigned skip connections and the extended decoders (27).

The Faster R-CNN we used is a 16-layer deep neural network of Ross B. Girshick et al. (28). Since Faster R-CNN highly integrates region generation, feature extraction, network training, target classification, and position regression into a whole (29), the accuracy is significantly improved compared to other algorithms. Yolov3 and Yolov5 are the real-time object detection algorithm of Redmon J et al. (30), respectively. As a real-time object detection algorithm, YOLOV series performs classification and bounding box regression in one step, making it much faster than most convolutional neural networks. For example, YOLO object detection is more than 1000x faster than R-CNN.

These models are selected to conduct a comparison between models with various model complexity as well as computational complexity.

### 2.4 Evaluation metrics

The performance of the classification network and the target detection network is measured by means of accuracy and AUC. ACC(Accuracy) indicates the percentage of the number of correctly classified samples to all the samples. AUC is the area under the ROC curve to evaluate the classification performance and target detection accuracy of the algorithm. The performance of the segmentation network is evaluated using IoU and Dice. IoU is a number from 0 to 1 that specifies the amount of overlap between the predicted and ground truth bounding box. The Dice coefficient is a statistic used to gauge the similarity of two samples. The performance of the target detection network is evaluated using mAP (Mean Average Precision) and AR(Average Recall). mAP is how much the ground truth bounding box overlaps with the predicted box. Recall is measuring the probability of ground truth objects being correctly detected as shown in **Table 2**.

**Table 2:** Evaluation Metrics.

Classification	**ACC**: Percentage of the number of samples correctly classified
	**AUC**: The area under the ROC curve
	**REC**: Recall Rate
Segmentation	**IoU**: The overlap rate of the predicted and ground truth bounding box
	**Dice**: the similarity of two samples
Target Detection	**mAP**: Mean Average Precision
	**AR :** Average Recall

## 3 Experiments

To provide proof for the proposed robustness evaluation we chose the challenging tasks of breast ultrasound segmentation classification and object detection. Each neural network is trained with 8617 breast ultrasound images containing benign and malignant base image data. In addition, to obtain reliable robust performance estimates, each training and testing run was repeated 100 times. Thus, all calculated metrics are averaged over 100 runs. The training details and results are described as follows.

### 3.1 Task-specific neural network

**Figure 3:**
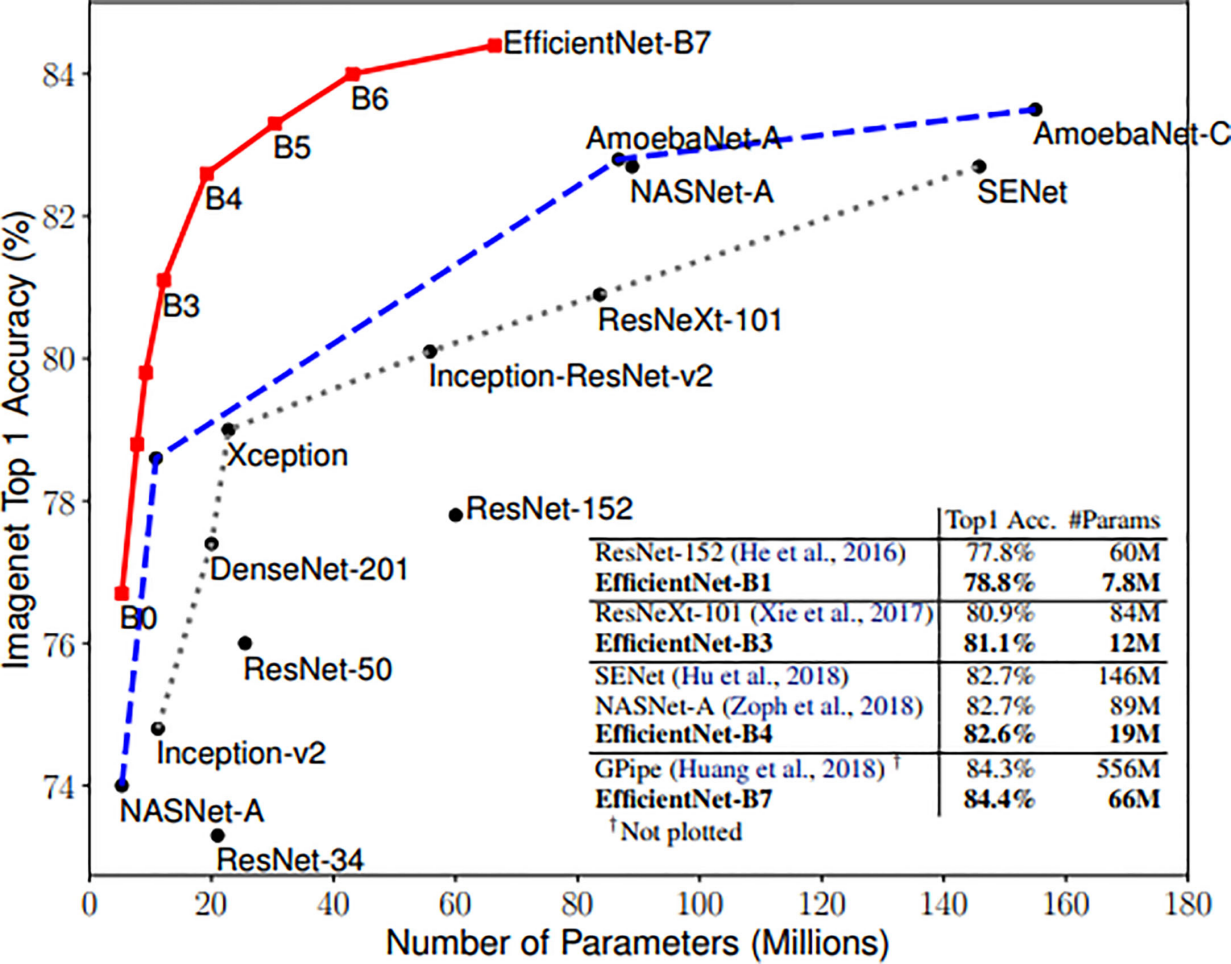
Model Size vs. ImageNet Accuracy (24).

Zhantao Cao et al. systematically evaluated the performance of several existing state-of-the-art classification methods for breast lesions CAD, including AlexNet, ResNet, and DenseNet (31). The comparison of networks was, however, not completed on noisy images. In this study, three neural networks, including ResNet, DenseNet, and EfficientNet, have been selected to Verify the robustness of the CAD system. ResNet and DenseNet are the most commonly used classification networks in the medical field (32). And the EfficientNet scaling method uniformly scales network width, depth, and resolution with a set of fixed scaling coefficients that can capture richer and more complex features. This feature can make it keep good robustness when the input picture is added noise. Compared with the existing CNNs, EfficientNet models achieve both higher accuracy and better efficiency (24), reducing parameter size and FLOPS by an order of magnitude. As shown in **Figure 3**.

In recent years, many researchers have employed convolutional neural networks (CNNs) to enable the automatic segmentation of medical images (33–35). Representative CNNs models include FCN (25), UNet (26), and SegNet (36). And in medical image segmentation for clinical applications, UNet based on a convolutional neural network is one of the most commonly used models (37). So we choose the following three segmentation networks. Therefore, UNet, UNet++ and FCN are selected for the medical image segmentation task. Zhantao Cao et al. systematically evaluated the performance of several object detection methods for breast tumor detection, including Fast R-CNN, Faster R-CNN, and YOLO. The comparison of networks was, however, not completed on noisy images. And we chose to evaluate three fully-convolutional deep architectures, namely Faster R-CNN, Yolov3, and Yolov5.

### 3.2 Image preprocessing

Every breast ultrasound image from our dataset was stored in an 8-bit PNG format. To feed the images in the training model, we changed grayscale PNG images to 3-channel. In our dataset, sizes of images differed from image to image. We unified the image size to be 512×512 pixels, for which we attempted to set a consistent training condition. Bilinear interpolation and cropping were used to resize images, and min-max scaling was applied to each image so that every pixel had a value in the range of 0-1. All the processing was performed using the opencv-python package.

### 3.3 Implementation details

First of all, these nine networks were pre-trained using ImageNet. Specifically, these networks were previously trained using for than 1000 000 images from the ImageNet database for classification, segmentation, or object detection (38). Then, they had been repurposed through a process of transfer learning. In transfer learning, the pre-trained model was able to transfer the knowledge such as weights and features gained from the source task is reused to a new, however similar classification, segmentation or target detection task with less computational resources and training time (39).

We used Adam and SGD optimizer to reduce loss. As the number of epochs increases beyond 100, training set loss decreases and becomes nearly zero. So we trained 100 epochs for each neural network. The training was conducted with a NVIDIA Tesla P40. Following the model training strategy presented in Sec. 2.2, each trained model is tested on the test set with two kinds of noise and their robustness is evaluated in terms of evaluation metrics.

### 3.4 Results

In this section, the results are demonstrated for image classification, image segmentation, and object detection separately.

#### 3.4.1 Robustness evaluation for classification

Regarding experiments without additional noise, no further processing is applied to raw breast ultrasound images, which are then used to refine the transfer learning hyperparameters for enhanced performance. Then the model obtained by transfer learning is tested on test sets with different levels of noise. With regards to the experiment with noise, different levels of Salt and Pepper Noise, Gaussian Noise, and Speckle Noise were added to raw breast ultrasound images, which are then used to refine the transfer learning hyperparameters for enhanced performance. Then the model obtained by transfer learning is tested on test sets with different levels of noise.

**Figure 4:**
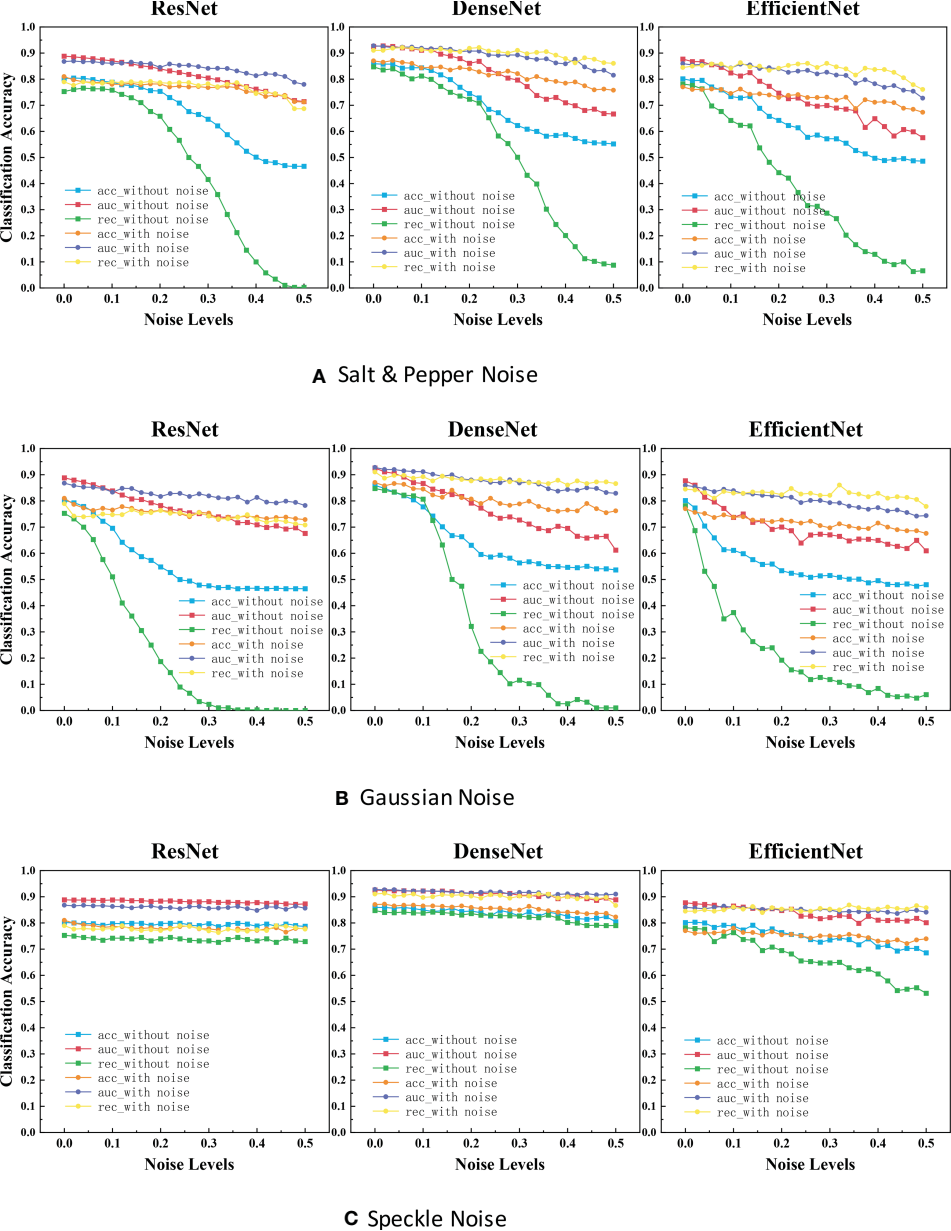
ACC scores and AUC scores for ResNet, DenseNet and EfficientNet on clean, noisy training images. **(A)** Salt & Pepper Noise. **(B)** Gaussian Noise.C. Speckle Noise.

Applying the above two experiments, the classification results for each noise are shown in **Figure 4**. The blue, red, and green lines represent the results of the first experiment, and the orange, purple, and yellow lines represent the results of the second experiment.

It is evident that the performance is reduced in all models due to noise. Referring to **Figure 4**, the most efficient model for Salt and Pepper Noise and Gaussian Noise is DenseNet, and the worst case is ResNet with its ACC scores dropped by approximately 40.0% and REC scores dropped by approximately 75.0%, which can be seen from the blue curve and green curve. The most efficient model for Speckle Noise is also DenseNet, and the worst case is EfficientNet with its REC scores dropped by approximately 25.0%, which can be seen from green curve. Speckle Noise has little effect on the performance of all networks. To our surprise, ResNet and DenseNet reveals better performance for Speckle Noise with its their performance has barely changed. This may be because unlike Gaussian or Salt and Pepper Noise, Speckle Noise is multiplicative noise.

#### 3.4.2 Robustness evaluation for segmentation

After using UNet, UNet++ and FCN to train the ultrasonic data set with and without noise, IoU scores and Dice scores on the test set for these three network structures were drawn as depicted in the figure below.

We can see that each family of networks exhibits a similar variation trend, despite different depths and model sizes. By considering the figure above, UNet, UNet++ and FCN reveals almost the same performance for images with added noise. However, FCN is slightly more robust as its range of segmentation accuracy decreases less. On the other hand, it can be seen that the segmentation accuracy of unet and unet++is almost exponentially decreasing due to the influence of noise, while the segmentation accuracy of fcn is almost linearly decreasing. Comparing the three figures, it is deducted that after the model is trained on the noisy data set, the segmentation accuracy on the test set remains almost unchanged as the noise concentration increases. It is deducted that using the original image and the image with noise to train plays a great role in improving the segmentation accuracy of each network.

#### 3.4.3 Robustness evaluation for target detection

With regards to the target detection, we used Faster R-CNN, Yolov3 and Yolov5 to train the ultrasonic data set with and without noise. The target detection results of each network with different levels of noise were drawn as depicted in the figure below.

As is shown in the figure, it is evident that Faster R-CNN is the most sensitive model to noise. We can see Yolov3 and Yolov5 perform signif icantly better than Faster R-CNN. Yolov3 and Yolov5 are slightly more robust as its range of segmentation accuracy decreases slower. That may be because YOLO makes predictions based on each complete image, so it implicitly encodes contextual information. There is no two-stage interception of ROI, so YOLO has a small background error. We were surprised to find that the performance of Faster R-CNN network drops dramatically close to zero when a little noise is added. Even after data enhancement, the performance of Faster R-CNN on noisy breast ultrasound data set is very poor. Observing the curve of Yolov3, we find that when the added noise level is greater than 0.2, it has high mAP and low AR. That means all predicted boxes were correct, but most of the ground truth objects were missed.

#### 3.4.4 Independent sample T-test

When we compared the results of these three experiments, we see that these curves have their own characteristics. In order to verify that the above experimental results were not simply due to chance, we can run a T-Test to see whether these experimental results are statistically significant. We randomly selected the model weights saved in the other 3 epochs to carry out the same experiment as above. The following results were obtained by t-test between the obtained new experimental values and the old experimental values. 

In the T-Test, like in most tests of significance, the significance threshold is traditionally set at p = 0.05. A p-value is basically the likelihood of finding a mean difference by chance if indeed there is no difference in the population (31). We can work out the chances of the result we have obtained happening by chance. From the **Tables 3**–**5**, you can see all P values were much higher than the p-value significance threshold of 0.05. This means our result is insignificant.

**Table 3:** T-Ttest results on salt and pepper noise.

Classification_ Salt and Pepper Noise				
P value Network	Pure_ACC	Pure_AUC	Fool_ACC	Fool_AUC
ResNet	.368	.735	.424	.719
DenseNet	.328	.157	.232	.271
EfficientNet	.247	.632	.886	.326
Segmentation_ Salt and Pepper Noise				
P value Network	Pure_IoU	Pure_Dice	Fool_IoU	Fool_Dice
U-Net	.288	.474	.623	.215
U-Net++	.334	.276	.426	.137
FCN	.256	.181	.102	.068
Target Detection_ Salt and Pepper Noise				
P value Network	Pure_AR	Pure_mAP	Fool_AR	Fool_mAP
Yolov3	.309	.378	.731	.718
Yolov5	.910	.908	.458	.447
Faster R-CNN	.880	.300	.818	.174

**Table 4:** T-test results on Gaussian noise.

Classification_ Gaussian Noise				
P value Network	Pure_ACC	Pure_AUC	Fool_ACC	Fool_AUC
ResNet	.154	.681	.384	.613
DenseNet	.299	.130	.845	.385
EfficientNet	.920	.135	.133	.110
Segmentation_ Gaussian Noise				
P value Network	Pure_IoU	Pure_Dice	Fool_IoU	Fool_Dice
U-Net	.320	.584	.706	.104
U-Net++	.621	.250	.347	.192
FCN	.248	.168	.080	.075
Target Detection_ Gaussian Noise				
P value Network	Pure_AR	Pure_mAP	Fool_AR	Fool_mAP
Yolov3	.971	.473	.247	.336
Yolov5	.919	.189	.216	.579
Faster R-CNN	.947	.322	.812	.108

**Table 5:** T-test results on Speckle noise.

Classification_ Speckle Noise				
P value Network	Pure_ACC	Pure_AUC	Fool_ACC	Fool_AUC
ResNet	.231	.674	.427	.528
DenseNet	.256	.356	.365	.675
EfficientNet	.762	.218	.349	.210
Segmentation_ Speckle Noise				
P value Network	Pure_IoU	Pure_Dice	Fool_IoU	Fool_Dice
U-Net	.418	.654	.421	.239
U-Net++	.235	.523	.456	.112
FCN	.211	.237	.123	.105
Target Detection_ Speckle Noise				
P value Network	Pure_AR	Pure_mAP	Fool_AR	Fool_mAP
Yolov3	.547	.331	.354	.428
Yolov5	.826	.293	.107	.284
Faster R-CNN	.768	.421	.652	.135

## 4 Discussion

### 4.1 General implications

Deep learning has achieved state-of-the-art performance in medical image classification, segmentation, and object detection tasks. However, ultrasound images obtained in adverse conditions are also vulnerable to noise that can affect the detection of diseases.

In the experimental results of our classification task (**Figure 4**), the segmentation accuracy of ResNet and EfficientNet trained with original images drops dramatically when a little noise is added to the test set. One reason for this behavior could be that medical images are highly standardized and small adversarial perturbations dramatically distort their distribution in the latent feature space (40, 41). ResNet has the highest classification accuracy and the strongest robustness after training with noisy images. Perhaps this is because ResNet has some deep adaptive capabilities. DenseNet is also robust when not training models with noisy images. But the disadvantage is that the segmentation accuracy is not very high. To our surprise, ResNet and DenseNet reveals better performance for Speckle Noise with its their performance has barely changed. This may be because unlike Gaussian or Salt and Pepper Noise, Speckle Noise is multiplicative noise.

**Figure 5:**
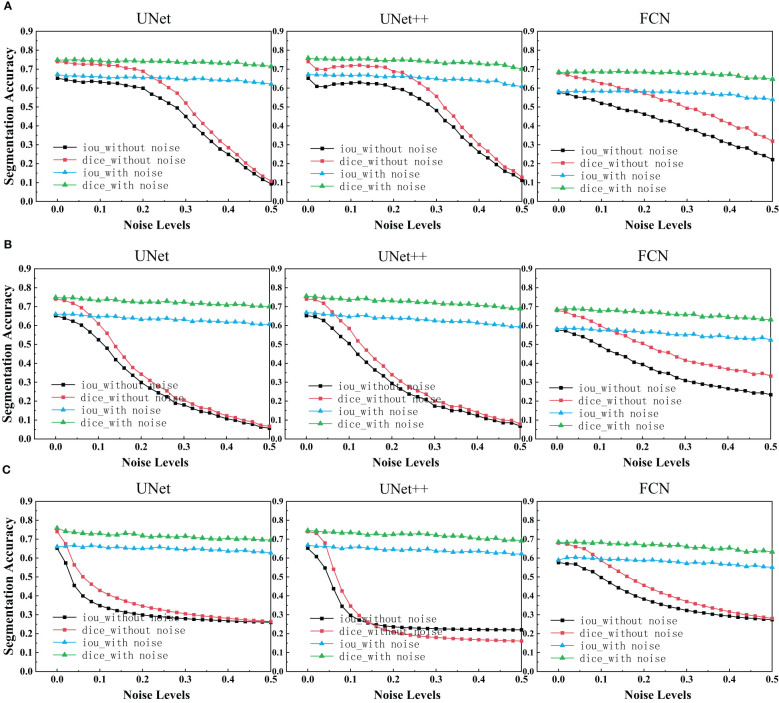
IoU scores and Dice scores for UNet, UNet++ and FCN on clean, noisy training images. **(A)** Salt & Pepper Noise. **(B)** Gaussian Noise. **(C)** Speckle Noise.

In the experimental results of our segmentation task (**Figure 5**), UNet and UNet ++ without using noise image training show great robustness to images with noise in the case of small noise level (<0.04). The segmentation accuracy of both of them decreases exponentially with the increase in noise concentration. However, the accuracy of FCN decreases linearly with the increase in noise concentration. Medical images obtained in clinical practice are easily accompanied by some tiny noise, so UNet and UNet ++ without using noise image training can be used in CAD systems. Additionally, training models with noisy images is effective at improving the overall robustness of the three segmentations. It is perhaps unsurprising that adding noise to breast ultrasound images of a trained network makes the network more robust and more general than a network trained only on clean data. This is because adding noise to training data is actually a recognized method to improve the robustness of deep networks and prevent overfitting (42, 43).

**Figure 6:**
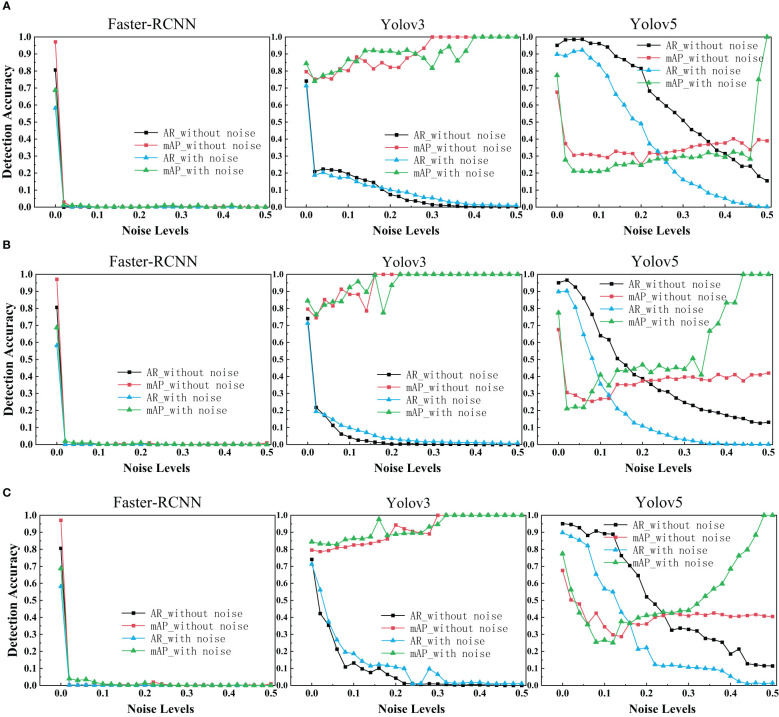
AR scores and mAP scores for Faster R-CNN, Yolov3 and Yolov5 on clean, noisy training images. **(A)** Salt & Pepper Noise. **(B)** Gaussian Noise. **(C)** Speckle Noise.

In the experimental results of our target detection task (**Figure 6**), We highlighted an interesting finding that Training neural networks with dataset mixed with noise images has no effect on improving the target detection accuracy of three networks. With regards to Faster R-CNN, it is surprising that the accuracy drops dramatically close to zero when a little noise is added. The exact reason needs further investigation. In addition, it is evident that the target detection performance is reduced in three models due to noise.

In deep learning, any noise is bound to mislead the learning process and subsequently, the learned feature may not be representative, and the determined parameters may not be optimal. Thus, providing sufficient high-quality data samples is essential for CAD systems.

### 4.2 Unique implications

In the classification task and object detection task, we found a common feature between them. The trend of these networks accuracy and noise level curves have unique characteristics. For example, in the experimental results of our classification task (**Figure 4**), the four-line segments of DenseNet are all relatively stable small wave shapes. While too small noise level makes the red line of ResNet drop significantly, increasing the noise concentration beyond a certain threshold (> 0.1), causing the model performance to remain stable. The red line trend of EfficientNet also drops sharply at first, but the difference between it and ResNet is that it fluctuated sharply when the noise concentration increases above a certain threshold (>0.1). This provides us an opportunity to predict the neural network architecture of a black-box model, by feeding it with different levels of noise and measuring the network accuracy.

On the other hand, the purpose of this study is to explore the impact of adding noise directly to the image on the performance of neural networks, which is different from the existing articles on robustness in the field of medical image processing. At present, most scholars study the robustness of CAD systems to adversarial noise, which makes them have to know the detailed network structure of CAD systems. In this paper, we can evaluate the robustness of CAD systems without knowing the network structure. Consequently, it provides a new way to evaluate the robustness of CAD systems in the future. In this study, the effects of noisy images on the performance of nine neural networks were quantified. This provides a useful foundation for designing more secure artificial intelligence aided diagnosis system.

## 5 Conclusion

In this paper, for the first time, we explored the robustness of a CAD system by adding different levels of noise directly in breast ultrasound images for the tasks of classification, segmentation, and detection. Two different experiments, including transfer learning without noise images and transfer learning with noise images, were utilized for assessing the effect of different noises on the performance of three medical imaging tasks. Our results show that using the dataset mixed with noise images for training has excellent advantages for image classification and segmentation tasks. In addition, there is a moderate to high impact on classification accuracy or segmentation accuracy when Salt and Pepper Noise, Gaussian Noise, or Speckle Noise is introduced to the images. The performance is reduced in all models due to noise. For classification tasks and segmentation tasks, ResNet and UNet++ have the best performance after transfer learning. As a result of classification tasks and target detection tasks, we find that the trend of these networks’ accuracy and noise level curves have unique characteristics. This finding provides us with a method to reveal the black-box architecture of CAD systems. With regards to detection tasks, We find that the performance of the Faster R-CNN network drops dramatically close to zero when a little noise is added. And the target detection performance of Yolov3 and Yolov5 trained with dataset mixed with noise images is worse than that trained with original images.

As an extension of this research, testing of these networks with other types of noise (such as Impulse, Erlang, and Rayleigh noise) and various types of noise combined on images presents a recommended future research opportunity. On the other hand, most deep CNNs have several similar network structures. The robustness of these combinations of different network structures is also a direction worth exploring. In the future, we also need to study the robustness issues caused by differences in network structure. In the study of the robustness of deep convolutional neural networks for medical diagnosis, most scholars only study the effect of adversarial noise on the performance of neural networks, which makes them have to know the detailed network structure of CAD systems. In this paper, we can evaluate the robustness of CAD systems without knowing the network structure. On the other hand, Chenyang Shen et al. investigate the effects of label noise on the deep learning–based computer-aided diagnosis model (18). They only trained and validated the CNN architecture for classification with seven noise levels of labels. However, the purpose of this study is to explore the impact of adding noise directly to the image on the performance of neural networks, which is different from the existing articles on robustness in the field of medical image processing. This research has opened a new direction for future research on evaluating the robustness of CAD systems.

## Data availability statement

The original contributions presented in the study are included in the article/supplementary materials. Further inquiries can be directed to the corresponding author.

## Ethics statement

The studies involving human participants were reviewed and approved by Department of Interventional Therapy, The Hwa Mei Hospital of the University of Chinese Academy of Sciences(Ningbo No.2 Hospital), Ningbo, China. The patients/participants provided their written informed consent to participate in this study.

## Author contributions

JJ: Conceptualization, methodology, software, visualization, investigation, formal analysis, writing - original draft. XJ: Visualization, software, writing - original draft. LX: Visualization, investigation. YZ: Data curation, collect data from hospitals, validation. DK: Writing - review & editing, supervision, resources. All authors contributed to the article and approved the submitted version.
